# Supplementary material for: Daytime temperature is sensed by phytochrome B in Arabidopsis through a transcriptional activator HEMERA
Source: Nat Commun. 2019 Jan 11;10:140. doi: 10.1038/s41467-018-08059-z (PMC6329817; doi:10.1038/s41467-018-08059-z)
Supplement: Supplementary file 1 — Supplementary Information [file 41467_2018_8059_MOESM1_ESM.pdf]

Supplementary Table 1. qRT-PCR primers for the genes examined in this study.

Accession	Gene name	Forward primer	Reverse primer
AT1G69960	*PP2A*	TATCGGATGACGATTCTTCGTGCAG	GCTTGGTCGACTATCGAATGAGAG
AT2G43010	*PIF4*	AACCAGATCATCTCCGACCGGTTT	TCCCGCCGGTGAACTAAATCTCAA
AT4G28720	*YUC8*	TGAAACAAAACAACCCACGA	TTGATTCGCTTTGGGTCTTC
AT3G15540	*IAA19*	ATCGGTGTGGCCTTGAAAG	AACATCCCCCAAGGTACATC
AT4G32280	*IAA29*	CACCATCATTGCCCGTATCA	CCACAGTAGCCGTTGTTGGA

Supplementary Table 2. Primers used for making the bait and prey constructs for the GST pulldown assays.

Accession	Gene name	Vector	Forward primer	Reverse primer
AT2G34640	*HMR*	pET42b	GGCGAATTCTATGGCGTCAATATCAACCACCAC	GGCCTGCAGTTAAGGATCAGTCTCCTCTTCAAAG
AT2G43010	*PIF4*	pCMX-PL2-NterHA	CGGAATTCATGGAACACCAAGGTTGGAG	GCAGCCCGGGCTAGTGGTCCAAACGAGAAC
AT2G43010	*PIF4-N1*	pCMX-PL2-NterHA	CGGAATTCATGGAACACCAAGGTTGGAG	GCAGCCCGGGCTAAGGTTGAACTCCGGGGAAC
AT2G43010	*PIF4-N2*	pCMX-PL2-NterHA	CGGAATTCATGGAACACCAAGGTTGGAG	GCAGCCCGGGCTATCGTTGGTTCGACTTGTTACC
AT2G43010	*PIF4-N3*	pCMX-PL2-NterHA	CGGAATTCATGGAACACCAAGGTTGGAG	GCAGCCCGGGCTAAGGGTCAGGACAGGCCTTAG
AT2G43010	*PIF4-C1*	pCMX-PL2-NterHA	CGGAATTCGATCATCATGAAGAAGCCCTAAG	GCAGCCCGGGCTAGTGGTCCAAACGAGAAC
AT2G43010	*PIF4-C2*	pCMX-PL2-NterHA	CGGAATTCCCTCCTCAAGTCATGCCTC	GCAGCCCGGGCTAGTGGTCCAAACGAGAAC
AT2G43010	*PIF4-C3*	pCMX-PL2-NterHA	CGGAATTCTCAGGATCAAACCGAAGGAG	GCAGCCCGGGCTAGTGGTCCAAACGAGAAC
