# Supplementary material for: Daytime temperature is sensed by phytochrome B in Arabidopsis through a transcriptional activator HEMERA
Source: Nat Commun. 2019 Jan 11;10:140. doi: 10.1038/s41467-018-08059-z (PMC6329817; doi:10.1038/s41467-018-08059-z)
Supplement: Supplementary file 4 — Reporting Summary [file 41467_2018_8059_MOESM4_ESM.pdf]

nature research

Corresponding author(s): Meng Chen

Last updated by author(s): Dec 11, 2018

Reporting Summary

Nature Research wishes to improve the reproducibility of the work that we publish. This form provides structure for consistency and transparency in reporting. For further information on Nature Research policies, see Authors & Referees and the Editorial Policy Checklist.

Statistics

For all statistical analyses, confirm that the following items are present in the figure legend, table legend, main text, or Methods section.

n/a	Confirmed	
☐	☒	The exact sample size (n) for each experimental group/condition, given as a discrete number and unit of measurement
☐	☒	A statement on whether measurements were taken from distinct samples or whether the same sample was measured repeatedly
☐	☒	The statistical test(s) used AND whether they are one- or two-sided<br>*Only common tests should be described solely by name; describe more complex techniques in the Methods section.*
☐	☒	A description of all covariates tested
☒	☐	A description of any assumptions or corrections, such as tests of normality and adjustment for multiple comparisons
☐	☒	A full description of the statistical parameters including central tendency (e.g. means) or other basic estimates (e.g. regression coefficient) AND variation (e.g. standard deviation) or associated estimates of uncertainty (e.g. confidence intervals)
☐	☒	For null hypothesis testing, the test statistic (e.g. F, t, r) with confidence intervals, effect sizes, degrees of freedom and P value noted<br>*Give P values as exact values whenever suitable.*
☒	☐	For Bayesian analysis, information on the choice of priors and Markov chain Monte Carlo settings
☒	☐	For hierarchical and complex designs, identification of the appropriate level for tests and full reporting of outcomes
☒	☐	Estimates of effect sizes (e.g. Cohen's d, Pearson's r), indicating how they were calculated

Our web collection on statistics for biologists contains articles on many of the points above.

Software and code

Policy information about availability of computer code

Data collection	No software was used.
Data analysis	No software was used.

For manuscripts utilizing custom algorithms or software that are central to the research but not yet described in published literature, software must be made available to editors/reviewers. We strongly encourage code deposition in a community repository (e.g. GitHub). See the Nature Research guidelines for submitting code & software for further information.

Data

Policy information about availability of data

All manuscripts must include a data availability statement. This statement should provide the following information, where applicable:

- Accession codes, unique identifiers, or web links for publicly available datasets
- A list of figures that have associated raw data
- A description of any restrictions on data availability

Arabidopsis mutants and transgenic lines as well as plasmids generated during the current study are available from the corresponding author on reasonable request. The source data of the gels and immunoblots in Figs 4c-e and 6d as well as the source data underlying Figs 1, 2b, 2d, 2e, 3b, 3d, 4a-b, 51-b, and 6b-c are provided in the Source Data file.

Field-specific reporting

Please select the one below that is the best fit for your research. If you are not sure, read the appropriate sections before making your selection.

☒ Life sciences ☐ Behavioural & social sciences ☐ Ecological, evolutionary & environmental sciences

For a reference copy of the document with all sections, see nature.com/documents/nr-reporting-summary-flat.pdf

Life sciences study design

All studies must disclose on these points even when the disclosure is negative.

Sample size	No sample-size calculation was performed. For hypocotyl measurements, as standard, at least 20 seedlings were used.
Data exclusions	No data were excluded from analysis.
Replication	At least three biological replicates were performed for hypocotyl measurements, transcript analyses, and immunoblots.
Randomization	For hypocotyl measurements, the seedlings were exposed to uniform light, seedlings were randomly picked for measurements.
Blinding	Not relevant to this study, because the mutants show clear phenotypes compared with the wild-type.

Reporting for specific materials, systems and methods

We require information from authors about some types of materials, experimental systems and methods used in many studies. Here, indicate whether each material, system or method listed is relevant to your study. If you are not sure if a list item applies to your research, read the appropriate section before selecting a response.

Materials & experimental systems

n/a	Involved in the study	
☐	☒	Antibodies
☒	☐	Eukaryotic cell lines
☒	☐	Palaeontology
☒	☐	Animals and other organisms
☒	☐	Human research participants
☒	☐	Clinical data

Methods

n/a	Involved in the study	
☒	☐	ChIP-seq
☒	☐	Flow cytometry
☒	☐	MRI-based neuroimaging

Antibodies

Antibodies used	Anti-PIF4 antibody, Agrisera, AS 12 1860; Anti-HA antibody, Sigma-Aldrich, H3663; anti-RPN6 antibody, Enzo Life Science, BML-PW8370-0100
Validation	The validation data for all antibodies can be found in the manufacturer's website.
